# Effects of Whole Corn Germ, a Source of Linoleic Acid, on Carcass Characteristics and Meat Quality of Feedlot Lambs

**DOI:** 10.3390/ani11020267

**Published:** 2021-01-21

**Authors:** Camila O. Nascimento, Douglas S. Pina, Luís G. A. Cirne, Stefanie A. Santos, Maria L. G. M. L. Araújo, Thomaz C. G. C. Rodrigues, William P. Silva, Mateus N. S. Souza, Henry D. R. Alba, Gleidson G. P. de Carvalho

**Affiliations:** 1 Department of Animal Science, Federal University of Bahia, Avenue Adhemar de Barros, 500, Ondina, Salvador 40170110, Brazil; douglaspinaufba@gmail.com (D.S.P.); stefanie_zootecnia@hotmail.com (S.A.S.); mariaaleonor@hotmail.com (M.L.G.M.L.A.); thomazguimaraes@yahoo.com.br (T.C.G.C.R.); willianzoo@yahoo.com.br (W.P.S.); mateusnetosilva@hotmail.com (M.N.S.S.); harrydoo@gmail.com (H.D.R.A.); 2 Institute of Biodiversity and Forestry, Federal University of Western, Vera Paz Street, Salé, Santarém 68040255, Brazil; lgabrielcirne@hotmail.com

**Keywords:** by-product, fatty acids, meat, nutrition, sheep

## Abstract

**Simple Summary:**

The industrialization of corn generates several by-products, including the whole corn germ (WCG). This, in turn, shows promise in diets for ruminants because it contains 85% of the total lipids that can increase the diets’ energy density (lipids naturally protected by the pericarp). Furthermore, WCG has around 56% linoleic acid (of total fatty acids), contributing to increased unsaturated fatty acid concentrations in meat. This research aims to evaluate the quantitative carcass traits and the quality of lambs’ meat supplemented with WCG to determine its optimum inclusion level in feedlot animals’ diet.

**Abstract:**

The whole corn germ (WCG), due to its desirable nutritional characteristics, has been studied as feed for ruminants. This study aimed to evaluate the effects of WCG inclusion as a linoleic acid source in diets for feedlot lambs on carcass characteristics, physicochemical composition, sensory attributes, and fatty acid profile of the meat. Forty non-castrated, crossbreed Dorper x Santa Inês lambs were distributed in a completely randomized design to evaluate the inclusion levels (0, 30, 60, 90, and 120 g/kg dry matter (DM)) of whole corn germ (WCG) in the diet. The dietary inclusion of WCG did not influence (*p* > 0.05) the weight gain and carcass characteristics, with the exception of the subcutaneous fat thickness (*p* < 0.01), which was higher in animals fed diets with higher levels of WCG. Lightness (L *; *p* = 0.04), yellowness (b *; *p* < 0.01), shear force (*p* = 0.04), linoleic fatty acid concentrations (*p* = 0.03), and total polyunsaturated fatty acids (*p* = 0.04) had a quadratic increase due to WCG inclusion in the diets. The use of up to 120 g/kg DM of WCG in lamb diets does not affect the carcass characteristics, physicochemical composition, and sensory attributes of the meat. Despite this, the best polyunsaturated fatty acid profile in lambs’ meat is obtained using 76.7 g/kg DM of WCG.

## 1. Introduction

Corn is one of the most used cereals in the world. Its industrialization process in the bioethanol production generates several by-products, among them distillers dried grains, whole corn germ (WCG), the outer seed shell, and oil [1,2,3]. These by-products can be used in animal and human feeding, biofuel and feedstock production, or other systems. The WCG is obtained from the wet degermination of corn grain by a mechanical extraction process [4].

The WCG has been studied in diets for ruminants due to its crude protein (10 to 15%) [5,6], ether extract (44%) [1], and linoleic acid (56% of total fatty acids) contents [1]. The inclusion of WCG in the ruminant diets aims to increase energy density [7] and polyunsaturated fatty acids, to obtain higher levels of conjugated linoleic acid (CLA) in the meat, which are beneficial to human health [8].

Given the importance of incorporating nutraceutical components in red meat, studies have been conducted to decrease the saturated fatty acid (SFA):polyunsaturated fatty acid (PUFA) ratio, maintaining the balance of the omega 6:omega 3 ratio [8]. Furthermore, it is aimed to increase the concentrations of CLA in the meat of ruminants [9]. The CLAs are biohydrogenation intermediates that, from the hydrogenation of linoleic acid by ruminal microorganisms, can pass from the rumen to be absorbed by the intestine. Subsequently, they are then incorporated into the meat of ruminants [10].

The WCG contains 85% of the total lipids in the grain [11], which is naturally protected by the pericarp. Therefore, this protection could decrease the biohydrogenation activity of ruminal bacteria on the unsaturated lipids present in the germ. Moreover, it increases the level of unsaturated lipids that will reach the intestine and, consequently, is incorporated into the meat [9].

To our knowledge, there are few studies with contradictory results of the dietary inclusion of WCG as a source of linoleic acid in diets for lambs. Given the nutritional characteristics, it is hypothesized that there is an inclusion level of WCG that increases diets’ energy density for feedlot lambs, improving performance, carcass yield, and quality due to the increase of the unsaturated fatty acid deposition in the meat.

This study aimed to evaluate the effects of whole corn germ inclusion as a linoleic acid source in diets for feedlot lambs on the carcass characteristics, physicochemical composition, sensory attributes, and fatty acid profile of the meat.

## 2. Materials and Methods

### 2.1. Location and Ethical Considerations

The experiment took place at the Experimental Farm of the Federal University of Bahia, located in the municipality of São Gonçalo dos Campos, Bahia, Brazil. This study was conducted in strict accordance with the recommendations presented in the Guide of the National Council for Animal Experimentation Control (CONCEA). The protocol was approved by the Ethics Committee on the Animal Use of the School of Veterinary Medicine and Animal Science at the Federal University of Bahia (Permit number: 70/2018).

### 2.2. Animals, Experimental Design, General Procedures, and Diets

Forty non-castrated, crossbreed Dorper × Santa Inês lambs with an average age of 4 months and an average initial body weight (BW) of 22.1 ± 4.0 kg (mean ± standard deviation) were distributed in a completely randomized design with five treatments and eight replicates (animals).

Lambs were housed in individual, covered stalls with suspended slatted wooden floors measuring 1.2 m^2^ (1.2 × 1.0 m), equipped with drinkers and feeding throughs. They received water ad libitum, and the experimental diets twice daily. Before the experiment began, all animals were identified and vaccinated (rabies and clostridial vaccines). They were then allocated at random to the treatments.

The lambs were kept in the feedlot for 75 days, which were preceded by a 15-day period of acclimation to the facilities, daily management, and diets. During this phase, they received diets composed of 500 g/kg of sorghum silage (*Sorghum bicolor* (L.) Moench) and 500 g/kg of concentrate mixture comprised of soybean meal, ground corn, WCG, urea, and a commercial mineral premix (Table 1).

The experimental diets consisted of 0, 30, 60, 90, and 120 g/kg WCG inclusion in diets (dry matter basis). The diets were formulated to supply the nutritional requirements of growing male lambs with a gain of 200 g/day as recommended by the National Research Council [12].

Animals were fed twice per day (09:00 h and 16:00 h), divided equally into two meals, as a total mixed ration (TMR). Nutrient intake was determined based on the difference between the amount of each nutrient contained in the feed offered and the feed refused during the experimental period. The amount of feed was adjusted daily, with an acceptable refusal amount about 10 to 20% of the total amount supplied to ensure ad libitum intake.

### 2.3. Chemical Analysis of Ingredients and Diets

During the feedlot period, samples of roughage, ingredients, diets, and refusals were weighed daily, harvested weekly, and subsequently frozen at −20 °C. At the end of the experimental period, the samples were then thawed, pre-dried in a forced-air oven at 55 °C for 72 h, and ground through a Wiley cutting mill with a 1-mm sieve. Ground samples were analyzed according to the methods of the Association of Official Analytical Chemistry [13] for dry matter (DM; method 934.01), ash (method 942.05), crude protein (CP = N × 6.25; method 968.06), and ether extract (EE; method 920.39) contents. The organic matter (OM) content of forage and feeds was determined by the following formula: OM (% DM) = 100 − ash (% DM).

The neutral detergent fiber was determined according to Mertens [14], using heat-stable alpha-amylase without the addition of sodium sulfite to the detergent, and acid detergent fiber (ADF) as described by Van Soest et al. [15]. Ingredients were also evaluated for lignin (method 973.18; AOAC) [16], by solubilization of cellulose with 72% (*w/v*) sulphuric acid.

The neutral (NDIP) and acid detergent insoluble protein (ADIP) contents were determined according to the methods of Licitra et al. [17]. Non-fibrous carbohydrates (NFC) contents were estimated according to Hall [18] and expressed in percentage.

### 2.4. Slaughtering Procedures and Carcass Characteristics

At the end of the experiment, animals were transported to a commercial slaughterhouse, subjected to a 16-h fasting period, and weighed to determine the final weight (FW). They were then stunned using the proper equipment to promote electronarcosis. Then, the animals were suspended, bled from the jugular vein and carotid artery before being skinned, and eviscerated according to the recommendations of procedures for handling and humane slaughter of the animals [19].

The mean pH was obtained by analyzing (in triplicate) the *Longissimus lumborum* (LL) muscles 45 min (initial) and 24 h after slaughter (final), using a digital HANNA skewer type HI 99163, connected to a penetration electrode, previously calibrated with pH 4.01 and 7.01 buffer solutions.

After the slaughter, carcasses were weighed to determine the hot carcass weight (HCW) and the hot carcass yield (HCY = HCW × 100/FW) and then transferred to a cold chamber (5 °C), where they remained for 24 h. Subsequently, the carcasses were weighed to determine the cold carcass weight (CCW) and cold carcass yield (CCY = CCW × 100/FW).

After the 24 h slaughtering period, the carcasses were subjectively evaluated for conformation, finishing, and fatness using a visual scale, from 0 to 5, as proposed by Cezar and Sousa [20], and the marbling of the meats. The carcass morphometric measurements were measured according to Osório et al. [21]. The measured parameters included the internal length, external length, leg length, leg circumference, rump width, chest width, chest depth, rump perimeter, and chest perimeter. The length and perimeter measures were taken using a tape measure, whereas those related to width and depth, with a manual meter aid.

The carcasses were cut longitudinally at the midline into two symmetrical antimeres. The carcass antimeres were sectioned between the 12th and 13th ribs to collect the loins (LL muscle) according to the methods described by Colomer-Rocher et al. [22]. Afterward, the loin eye area (LEA) was assessed using plastic transparency sheets and an appropriate pen. Thus, the following measures were established: the length and the maximum depth of the LL muscle, in cm, measured with the aid of a ruler and calculated from the ellipse formula: LEA = (length/2 × depth/2) π, in cm^2^, proposed by Silva Sobrinho [23].

The subcutaneous fat thickness (SFT) in the carcasses was measured, in mm, with the aid of a digital caliper at ¾ distance from the medial side of the LL muscle, to the side of the spinous process. Subsequently, loins from the left and right sides were collected from each animal and immediately weighed, deboned, identified, vacuum packed in polyethylene packs, and stored at –20 °C for further evaluation of physicochemical analysis, sensory attributes, and fatty acid profile.

### 2.5. Meat Physicochemical and Sensory Analysis

Meat analysis was performed after thawing the loins in plastic bags (10 °C for 12 h). The samples were then dissected with the aid of a scalpel and knife. The color parameters were evaluated using the left side of the loins collected from the lambs. The color parameters were determined with the aid of a Minolta CR-400 colorimeter, using the CIELAB (Commission Internationale de l’Eclairage L, a*, b*) system through the coordinates of lightness (L *), redness (a *), and yellowness (b *). The colorimeter was calibrated with a white ceramic plate and illuminant C, 10°, for standard observation, and it was operated using an open cone.

Evaluation of meat color was carried out after the myoglobin was oxygenated by exposing the LL to the atmosphere for five minutes [24]. Then, as described by Miltenburg et al. [25], the L *, a *, and b * coordinates were measured at three different points on the muscle surface, and subsequently averaged in triplicate for each coordinate per animal.

Cooking weight losses (CWL) of LL muscle were measured in each loin sample with 1.5 cm thickness, 3.0 cm length, and 2.5 cm width cubic samples (in triplicate), free of visible connective tissue. Raw samples were weighed, placed in an aluminum-coated tray, and cooked in a preheated oven at 170 °C until the center reached 70 °C, measured using a copper-constantan thermocouple equipped with a digital reader. Subsequently, samples were cooled at room temperature and weighed again. The cooking weight loss of each sample was obtained as the difference between the weights before and after cooking [26].

The Warner–Bratzler shear force (WBSF) analyses were determined using the same cooked meat samples used to measure cooking losses. At least three cores 25 mm in diameter × 25 mm in length were removed from each sample. The WBSF was measured by a texture analyzer (Texture Analyzer TX-TX2; Mecmesin, Nevada, United States) fitted with a Warner–Bratzler-type shear blade according to the standard procedure described by Wheeler et al. [27]. The WBSF values were expressed in kgf/cm^2^.

Evaluation of the proximate composition was carried out using the samples of LL muscles (in natura), which were lyophilized for 72 h. They were then ground using a ball mill and analyzed for moisture, ash, protein, and total lipids contents according to the methods described by the AOAC [13].

The LL samples used in the sensory characteristics were evaluated using an unstructured hedonic scale of nine points by 100 untrained panelists. All panelists included 61 women and 31 men in an age group between 19 and 50 years of age accustomed to eating lamb meat. The samples were cooked on an electric grill (George Foreman Grill Jumbo GBZ6BW model) with the aid of a digital thermocouple. The thermocouple was inserted in the geometric center of each sample to monitor the temperature of each steak, which was cooked until the geometric center reached 75 °C. After cooking, the samples of LL muscle of lambs fed different WCG inclusion levels (0, 30, 60, 90, and 120 g/kg) were then cut into cubes. Afterward, they were transferred to encoded pre-heated beakers, which were placed in a water bath (75 °C) and covered with aluminum foil to ensure minimum heat loss and aroma volatiles.

The tests were carried out between 09:00 and 12:00 h, and consumers were placed in individual cabins. During the sensory evaluation, each taster was provided two samples per treatment without salt or condiments in plastic containers with coded lids; each taster also received water and cream cracker-type biscuits for intake between tastings to remove the residual flavor.

The sensory attributes of the LL muscle were evaluated using an affective method on a structured hedonic scale. The tasters evaluated the following attributes: taste, tenderness, juiciness, aroma, and overall acceptance. The scores ranged from 1 to 9, as follows: 1, disliked very much; 2, disliked; 3, moderately disliked; 4, slightly disliked; 5, indifferent; 6, slightly liked; 7, moderately liked; 8, liked; and 9, liked very much). The intensities of the lamb meat flavor and aroma characteristics were also evaluated according to American Meat Science Association (AMSA) [28].

### 2.6. Fatty Acid Profile

The composition of lipids extracted from the samples of diets and LL was determined by converting the lipid extracts to fatty acid methyl esters (FAMEs). Afterward, FAMEs were prepared following the methodology described by O’Fallon et al. [29].

Meat samples (in natura) were ground (homogenized) in grinder (Cadence 150W MDR 302), lyophilized for five days, and milled (homogenized) again. Approximately 0.5 g of dry sample was placed in a 16 × 125 mm pyrex culture tube, which contained 1.0 mL of internal standard C19:0 (189-19 Sigma Aldrich, São Paulo, Brazil; 10 mg of C19:0/mL of MeOH), added with 0.7 mL of 10 N KOH in water, 5.3 mL of MeOH. The tubes were incubated at 55 °C in a water bath for 1 h 30 min with vigorous stirring to permeate every 20 min to dissolve and hydrolyze the sample. After cooling in an ice-water bath, 0.58 mL of 24N H_2_SO_4_ in water was added. The contents of the tubes were mixed by shaking and precipitated with K_2_SO_4_. They were then incubated in a water bath at 55 °C for 1 h 30 min with shaking for 5 s every 20 min.

After synthesizing the FAMEs, the tubes were cooled in an ice-water bath. Subsequently, 3 mL of hexane was added, and the contents of the tubes were mixed for 5 min using a vortex. Subsequently, tubes were immediately centrifuged for 5 min, and supernatant with hexane containing FAME was transferred to gas chromatography vials. The vials were capped and placed at −20 °C until analyses.

The FAME composition was determined using a gas chromatograph (SPTM-2560) column (100 m–25 mm–0.2 μm pore size) with a flame ionization detector and split injector (Thermo Scientific Inc.) and the hydrogen as the carrier gas (1 mL min^−1^). Nitrogen was used as the auxiliary gas. The temperature of the injector and detector was 250 °C at a 15:1 split. The initial temperature of the oven was adjusted to 70 °C; this temperature was maintained for 4 min, and gradually increased (at 13 °C/minute) until reaching 175 °C, where it remained for 27 min. Then, it was increased (at 4 °C/minute) until reaching 215 °C, which was maintained for 31 min [30].

Identification of FAs was performed comparing the retention times of FAME to those of the standards (37 Component FAME Mix from Supelco Inc., Sigma Aldrich Darmstadt, Germany) and published chromatograms [31,32]. The quantification of FAMEs was conducted based on the equation proposed by Sukhija and Palmquist [33]: ((total area of the peaks - area of the internal standard)/area of the internal standard) × (concentration of the internal standard/weight of the lyophilized sample)). Fatty acid profile was expressed in milligrams of fatty acids per kg of meat (mg/kg).

The group sums, ratios, and total contents of the saturated fatty acids (SFAs), monounsaturated fatty acids (MUFAs), and polyunsaturated fatty acids (PUFAs), and the MUFA:SFA, PUFA:SFA, PUFA:MUFA, and n-6/n-3 values were calculated from the identified FA profiles.

The desirable fatty acids (DFA) were calculated according to Rhee [34]. The activity indexes of the elongase and Δ^9^-desaturase enzymes for FAs with 16 and 18 carbons were determined using the methodology proposed by Malau-Aduli et al. [35] and Kazala et al. [36].

The nutritional quality of the lipid fraction of the LL muscle, the atherogenicity (AI), and thrombogenicity (TI) indexes were calculated as proposed by Ulbricht and Southgate [37]. The ratio of hypocholesterolemic:hypercholesterolemic (h:H) fatty acids ratio, as well as the concentrations of hypercholesterolemic, neutral, and hypocholesterolemic fatty acids, were evaluated and adapted according to Bessa [38] and Santos-Silva, Bessa, and Mendes [39]. 

### 2.7. Statistical Analyses

The experiment was conducted as a completely randomized design with five treatments and eight replicates (lambs) per treatment. The following statistical model was used:

Ŷij = μ + NLi + εij(1)

where Ŷij = the observed value in the portion that received the treatment i in repetition j; μ = the overall mean; NLi = the fixed effect of the inclusion level of whole corn germ (i = 0, 30, 60, 90, and 120 g/kg); and εij = the effect of experimental error associated with each presupposition observation normal independent distribution (NID) ~ (0, σ2).

The data were subjected to analysis of variance and regression testing, with the freedom degrees evaluated by linear or quadratic effects. The command PROC MIXED of SAS (version 9.4, SAS Institute Inc.) was used to estimate the linear or quadratic parameters of significant models. To quadratic models, the maximum or the minimum point were obtained by making the second derivative of the quadratic model equal to zero.

The data related to sensory analysis were submitted to statistical analysis considering the WCG inclusion levels as a fixed effect and panelists as a random effect. The Poisson distribution was analyzed using the PROC GLIMMIX procedure of SAS 9.4. The variables were evaluated adopting 0.05 as the critical level of probability for type-I error.

## 3. Results

### 3.1. Intake and Carcass Characteristics

Total dry matter intake decreased (*p* < 0.01), whereas EE intake (*p* < 0.01) increased as WCG was included in the diets (Table 2).

No effect was observed from the inclusion of WCG (*p* > 0.05) on the FW, subjective evaluation (conformation, finishing, and marbling), carcass yields, and LEA of the lambs (Table 2). However, the inclusion of WCG had a quadratic effect on SFT (*p* < 0.01), with a minimum value of 2.31 mm observed at the WCG level of 46.9 g/kg DM.

The inclusion of WCG in the diet of lambs did not influence (*p* > 0.05) the morphometric measurements, with the exception of leg circumference (*p* = 0.01) and chest depth (*p* = 0.01), which decreased linearly (Table 2).

### 3.2. Physicochemical Composition and Sensory Attributes of the Meat

The dietary inclusion of WCG had a quadratic effect on lightness (L *; *p* = 0.04), yellowness (b *; *p* < 0.01), and WBSF (*p* = 0.04) (Table 3) with the maximum values of 39.6, 6.9, and 3.2 kgf/cm^2^, estimated at WCG levels of 63.0, 67.0 g/kg and 55 g/kg DM respectively.

The inclusion of WCG did not influence (*p* > 0.05) the proximate composition and sensory attributes of the LL muscle of lambs (Table 3).

### 3.3. Meat Fatty Acid Profile

The concentrations of the fatty acids in the LL muscle of lambs did not change (*p* > 0.05) with WCG inclusion in the diet (Table 4). However, the inclusion of WCG had a quadratic effect in the concentration of linoleic fatty acid (C18:2 n-6) (*p* = 0.03), with a maximum value of 1408 mg/kg, estimated at a WCG level of 76.7 g/kg.

The inclusion of WCG in the lambs’ diet had a quadratic effect on the total polyunsaturated fatty acids (PUFAs) (*p* = 0.04), with a maximum value observed of 2072 mg/kg, estimated at WCG levels of 71.8 g/kg DM and linear effect on the omega-6:omega-3 ratio (*p* = 0.03) (Table 5). However, no effect (*p* > 0.05) was observed from the inclusion of WCG on the other fatty acid sums, enzymatic activities of Δ9-desaturase and elongase, as well as the levels of nutritional quality of the lipid fraction related to human health.

## 4. Discussion

### 4.1. Nutrient Intake and Carcass Characteristics

The decrease of DM intake can be related to the chemostatic regulation of voluntary intake due to the increased EE intake and its content in the diets [40]. The WCG used in the current study had 41.2% EE in its composition. Consequently, there was a range of EE content in the experimental diets from 3.1 to 7.6%. Despite being a good energy source, EE levels above 5% in ruminant diets can be toxic to ruminal microorganisms [41].

The DM intake observed in the current study is in agreement with the results previously reported by Urbano et al. [3]. According to the authors, there was a decrease in lambs’ nutrient intake when WCG totally replaced ground corn. The diet tested by them reached values of up to 12% EE.

According to De Souza et al. [42], higher ATP and metabolizable energy are obtained through long-chain fatty acids from the diet in comparison to short-chain fatty acids from ruminal digestion. The WCG inclusion in lambs’ diets did not influence the FW and the quantitative characteristics of the carcasses. These results can be explained and related to the higher intake of lipids and, consequently, available metabolizable energy. In this way, there was an efficient use of the protein for muscle growth [43]. Therefore, the animals maintained similar performance levels, even with the reduction of dry matter intake.

Although the quadratic effect obtained for the SFT of the carcasses does not have a biological explanation, the higher energy input due to the dietary inclusion of WCG contributed to the increase of this parameter, which was 3.3 mm in the highest level of WCG tested.

The higher diets’ energy density with the WCG inclusion contributed not only to the animals’ energy requirements and may have promoted higher finishing of the carcasses [44]. This may have also favored, consequently, the preservation of meat quality, which was confirmed with the lowest WBSF values in the meat of these animals (Table 3).

### 4.2. Physicochemical Composition and Sensory Attributes of the Meat

The mean pH values of meat were outside the recommended values reported by Sañudo et al. [45] for sheep meat, which is 6.56 to 6.69 for the initial pH, and from 5.66 to 5.78 for the final pH. Nevertheless, the lamb meat showed good quality and was not classified as dark, firm, and dry (DFD) or pale, soft, and exudative (PSE).

The pH varies according to the biochemical process of the transformation of muscle to meat. The glycogen, energetic muscle conductor, is converted into lactic acid, decreasing the pH of the meat. The animals in the current study were submitted to a period of pre-slaughter fasting (16 h of fasting for weighing and sending the animals to the slaughter).

When glycogen stores in muscle before slaughter decrease, it is likely to expect a higher ultimate pH and sometimes DFD meat. This characteristic is related to factors such as the animal age, inadequate nutrition, handling procedures before slaughter, transportation time to the slaughterhouse, long fasting period, and animal behavior, among others. Controlling these factors ensures necessary amounts of glycogen (57 µmoles/g of muscle), essential for post-mortem muscle acidification [46].

Meat color can be influenced by several factors, including diet, intramuscular fat content, and pH values [47]. The high final pH (above 6.0) makes mitochondrial cytochrome oxidases more active. Thus, increased oxygen consumption may increase the concentration of deoxygenated myoglobin, resulting in dark-colored meats. In addition, the higher pH contributes to higher water retention capacity, making the meat paler [48]. In the current study, despite having a higher pH value after 24 h, the results observed for color coordinates were not far from the values reported by Leão et al. [49] (L * = 45.68; a * = 15.17; b * = 4.93) in the sheep meat.

The results observed for color parameters of lightness (L *) and the yellowness (b *) can be related to the fat deposition in the meat. These findings are in agreement with the results reported by Fruet et al. [50]. They reported that higher values for lightness and yellowness in the meat of ewe fed diets exclusively with grains. Moreover, the carotenoid content deposited in the intramuscular fat of these animals’ meat [51], from ground corn [52], can promote higher intensity of yellowness in fat. In turn, there is an increase in the luminosity of meat [53].

### 4.3. Fatty Acid Profile

In the current study, the most representative fatty acids in the LL muscle of lamb meat were oleic (C18:1 n-9), followed by the palmitic (C16:0), and stearic (C18:0) fatty acids. The same result was observed previously in other studies when the fatty acid profile was evaluated in lambs’ meat [9,54,55,56]. The concentration of linoleic fatty acid (C18:2 n-6) in lambs’ meat was an important finding in the current study. The inclusion of 76.7 g/kg of WCG provided the highest value in the lambs’ meat (1408 mg/kg of meat). The increase in C18:2 n-6 content in the meat of lambs fed with this dietary inclusion level of WCG may also be directly related to the content of this fatty acid in diets (Table 1).

The concentration of C18:2 n-6 was high in the WCG used in the experimental diets, and a part of this fatty acid, in the materials’ matrix, may have escaped from ruminal biohydrogenation [57]. This result is in agreement with the report of Urbano et al. [8], who mentioned higher levels of C18:2 n-6 and CLA (C18:2 9c 11t) fatty acids in the lambs’ meat when animals were fed diets with total replacing of ground corn by WCG.

Both in humans and for animals, the C18:2n-6, as well as alpha-linolenic acid (C18:3n-3), precursors of the omega-6 and omega-3 long-chain fatty acid groups, are necessary to maintain cell membranes, brain functions, and transmission of nerve impulses under normal conditions [58]. Moreover, the C18:2n-6 fatty acid acts as precursor to conjugated linoleic acids (CLAs), known for their health benefits, especially the reduction of body fat, which makes it an influential role in the control of chronic non-communicable diseases [59].

Oils and vegetable by-products composed of high amounts of fat are used in ruminant diets to improve milk and meat fat quality [1]. This use is supported by the fact that these oilseeds’ lipid profile has high rates of essential fatty acids, such as C18:1, C18:2 n-6, and C18:3 n-3 [60].

Saoussem et al. [61] evaluated the fatty acid profile of WCG and its main parts (germ, pericarp, and endosperm). They observed that the germ concentrates about 65.4% of C18:2 n-6 of the total fatty acids. In the current study, the higher C18:2 n-6 content contributed to the increase in total PUFA in the lambs’ meat. Moreover, it is a fatty acid of the n-6 family and, consequently, increased these fatty acids sums. Nevertheless, the n-6:n-3 ratio remained within the recommended value proposed by the Food and Agriculture Organization (FAO) [62] for human health (n-6:n-3 from 5:1 to 10:1).

## 5. Conclusions

Whole corn germ can be used up to 120 g/kg DM in the total diet for feedlot lambs without causing negative impact. Carcass and meat quality changes based on the quantitative characteristics, physicochemical composition, and sensory attributes of the meat.

The inclusion of WCG in lambs’ diets decreased dry matter intake. In this way, its inclusion increased the feeding efficiency and the possibility of better economic results because a smaller amount of feed was included in the diets during the feedlot period.

Nevertheless, if the meat industry seeks a better quality product, under the nutritional aspect, the level of 76.7 g/kg DM of WCG provides higher concentrations of polyunsaturated fatty acids in the meat, especially the linoleic acid that contributes to the increase of fatty acids beneficial to human health.

## Figures and Tables

**Table 1:** Proportion of ingredients, chemical composition, and fatty acid profile of the experimental diets and whole corn germ (WCG).

Item	Whole Corn Germ (g/kg DM)					WCG
	0	30	60	90	120	
	**Ingredient proportion (g/kg DM)**					
Sorghum silage	500	500	500	500	500	-
Ground corn	330	303	276	248	221	-
Soybean meal	145	142	139	137	134	-
Whole corn germ	0	30	60	90	120	-
Urea	10	10	10	10	10	-
Mineral supplement ^1^	15	15	15	15	15	-
	**Chemical composition (g/kg as-fed)**					
Dry matter (DM)	594	595	596	596	597	920
	**Chemical composition (g/kg DM)**					
Organic matter	843	844	845	846	847	911
Mineral matter	37	37	37	37	36	93
Crude protein	175	175	174	174	174	137
Ether extract	31	42	54	65	76	414
Neutral detergent fiber	394	402	409	416	423	418
Acid detergent fiber	198	203	208	213	218	208
NIDP ^2^	7.6	7.4	7.3	7.2	7.0	11
ADIP ^3^	3.8	3.7	3.7	3.7	3.6	3.0
Cellulose	96	100	104	108	112	159
Hemicellulose	196	198	200	202	204	210
Lignin	102	103	104	105	106	48
Non-fibrous carbohydrates	360	343	325	307	289	21
	**Fatty acid profile (mg/kg of diet)**					
Total	21,877	33,484	45,091	56,688	68,295	434,910
Caprylic (C8:0)	4.0	5.0	6.0	6.0	7.0	2.0
Capric (C10:0)	36	41	47	52	57	247
Lauric (C12:0)	50	55	60	66	71	287
Myristic (C14:0)	419	575	730	885	1041	6035
Palmitic (C16:0)	3392	4924	6457	7989	9522	58,344
Palmitoleic (C16:1)	81	103	125	146	168	875
Stearic (C18:0)	724	1016	1309	1601	1894	11,181
Oleic (C18:1 n-9)	5848	9937	14,026	18,108	22,197	150,108
Linoleic (C18:2 n-6)	9974	15,079	20,183	25,283	30,387	192,030
α-linolenic (C18:3 n-3)	517	599	680	763	844	3485

^1^ Assurance levels (provided per kilogram of active elements): zinc—3.800 mg, sodium—147.00 g, manganese—2.000 mg, cobalt—15.00 mg, copper—590.00 mg, sulfur—20.00 g, iodine—50.00 mg, chromium—20.00 mg, molybdenum—300.00 mg, calcium (min)—110.00 g, calcium (max)—135.00 g, and fluorine (max)—87.00 mg. ^2^ Neutral detergent insoluble protein. ^3^ Acid detergent insoluble protein.

**Table 2:** Average nutrient intake during the experimental period, quantitative, subjective, and carcasses morphometric measurements of feedlot lambs fed diets with whole corn germ (WCG).

Item	Inclusion Level of WCG (g/kg DM)					SEM	*p*-Value ^1^	
	0	30	60	90	120		L	Q
**Total intake (kg)**								
Dry matter	72.9	70.6	64.7	55.2	59.2	2.26	<0.01	0.30
Crude protein	13.8	13.7	13.6	13.7	13.7	0.06	0.15	0.20
Ether extract	2.5	3.5	4.4	5.4	6.4	0.01	<0.01	0.27
**Weights (kg)**								
Initial weight	26.9	28.0	26.5	25.8	25.8	-	-	-
Final weight	42.7	43.5	42.1	41.8	41.5	0.71	0.07	0.64
Hot carcass weight	18.2	17.9	18.0	18.0	18.1	0.63	0.99	0.68
Cold carcass weight	17.7	17.4	17.5	17.5	17.6	0.62	0.97	0.77
**Yields (%)**								
Hot carcass yield	42.5	41.5	43.0	42.9	42.0	1.13	0.98	0.75
Cold carcass yield	41.3	40.3	42.0	41.8	40.7	1.10	0.96	0.63
**Subjective evaluation**								
Conformation	3.0	2.7	3.0	2.9	2.8	0.12	0.92	0.95
Finishing	2.7	2.6	2.6	2.3	2.8	0.14	0.94	0.08
Marbling	2.3	2.1	2.1	2.2	2.2	2.12	0.95	0.56
**Morphometric measurements (cm)**								
External length	54.5	55.0	53.5	55.1	53.8	1.02	0.74	0.91
Internal length	56.2	57.6	55.2	55.8	54.2	1.47	0.24	0.61
Leg length	36.5	35.8	35.5	36.6	34.4	0.83	0.22	0.61
Leg circumference	44.6	44.1	44.8	44.1	41.2	0.81	0.01	0.04
Chest width	25.4	24.6	24.6	24.0	24.7	0.68	0.36	0.41
Rump width	20.4	20.2	20.4	20.0	20.2	0.63	0.77	0.94
Chest depth	26.8	25.3	24.8	25.3	24.2	0.58	0.01	0.44
Rump perimeter	52.5	52.0	53.8	53.2	54.5	1.08	0.18	0.79
***Longissimus lumborum* muscle**								
Loin eye area (cm^2^)	10.9	10.9	10.4	11.5	11.5	0.73	0.50	0.61
Subcutaneous fat thickness (mm)	2.9	2.3	2.3	2.3	3.3	0.25	0.31	<0.01

^1^ L = linear; Q = quadratic; significant at *p* < 0.05.

**Table 3:** Physicochemical composition, and sensory attributes of the *Longissimus lumborum* muscle of feedlot lambs fed diets with whole corn germ (WCG).

Item	Inclusion Level of WCG (g/kg DM)					SEM	*p*-Value ^1^	
	0	30	60	90	120		L	Q
pH 0 h	6.8	7.0	6.6	6.7	7.0	0.10	0.67	0.11
pH 24 h	6.4	6.0	5.8	6.1	6.0	0.18	0.27	0.13
Color parameter								
Lightness (L *)	36.5	38.7	39.9	39.0	37.1	1.16	0.69	0.04
Redness (a *)	19.4	20.6	20.0	20.5	20.2	0.38	0.27	0.19
Yellowness (b *)	5.0	6.3	7.7	6.8	6.0	0.45	0.10	<0.01
Shear force (kgf/cm^2^)	2.7	2.8	3.4	2.9	2.5	0.26	0.60	0.04
Cooking weight loss (%)	20.8	18.8	20.0	17.1	18.8	2.26	0.42	0.50
**Chemical composition**								
Moisture	73.8	73.6	73.5	73.2	73.7	0.38	0.64	0.40
Ash	1.1	1.1	1.1	1.1	1.1	0.02	0.45	0.27
Crude protein	22.0	21.2	22.0	22.2	21.1	0.34	0.56	0.29
Ether extract	3.0	3.8	3.1	3.4	3.3	0.31	0.69	0.46
**Sensory attributes**								
Flavor	6.3	6.5	6.3	6.5	6.5	0.28	0.68	0.90
Tenderness	7.7	7.5	7.1	7.6	7.7	0.28	0.96	0.41
Juiciness	7.3	7.1	6.7	7.3	7.3	0.27	0.95	0.42
Aroma	6.6	6.9	6.7	7.1	6.7	0.26	0.62	0.56
Overall acceptance	6.9	7.0	6.7	7.0	7.0	0.27	0.73	0.72

^1^ L = linear; Q = quadratic; significant at *p* < 0.05.

**Table 4:** Fatty acid composition (mg/kg of meat) of the *Longissimus lumborum* muscle of feedlot lambs fed diets with whole corn germ (WCG).

Item	Inclusion Level of WCG (g/kg DM)					SEM	*p*-Value ^1^	
	0	30	60	90	120		L	Q
	Saturated fatty acids (SFA)							
Caprylic (C8:0)	11.7	15.0	17.5	15.0	17.4	2.31	0.16	0.46
Capric (C10:0)	67.3	61.8	49.3	65.8	66.2	9.37	0.95	0.27
Lauric (C12:0)	42.7	38.7	33.8	48.1	46.8	5.95	0.38	0.30
Myristic (C14:0)	625	575	582	586	617	74.4	0.98	0.58
Pentadecanoic (C15:0)	87.6	95.2	79.7	73.9	77.8	15.43	0.43	0.96
Palmitic (C16:0)	7023	6459	6936	5917	6557	732	0.55	0.75
Heptadecanoic (C17:0)	316	260	308	296	269	21.98	0.42	0.97
Stearic (C18:0)	5098	4652	6153	4952	5039	375	0.88	0.27
Arachidic (20:0)	36.8	33.1	44.0	36.1	37.3	2.61	0.63	0.39
Behenic (22:0)	97.3	72.0	91.9	81.3	77.4	5.83	0.13	0.59
	Branched-chain fatty acids (BCFA)							
Iso—14:0	8.5	6.1	10.8	8.4	8.2	1.20	0.66	0.58
iso—15:0	35.3	26.1	31.6	28.8	24.0	5.80	0.31	0.98
anteiso—15:0	37.6	28.5	42.4	36.6	30.1	4.71	0.66	0.43
iso—16:0	46.6	34.9	56.1	43.9	39.4	4.76	0.73	0.31
iso—17:0	7.2	6.3	5.0	5.4	4.5	1.07	0.09	0.66
anteiso—17:0	6.8	5.2	5.8	5.2	7.6	1.16	0.67	0.14
iso—18:0	5.8	6.5	7.2	6.4	6.7	0.60	0.39	0.37
	Monounsaturated fatty acids (MUFA)							
Miristoleic (C14:1)	23.2	23.5	18.9	19.5	20.3	3.76	0.43	0.67
Palmitoleic (C16:1)	605	544	531	366	495	83.21	0.17	0.48
C18:1 trans-9	5.4	6.6	9.3	6.2	8.4	3.12	0.60	0.78
C18:1 trans-10	213	201	252	216	222	19.46	0.62	0.50
Vaccenic (C18:1 trans-11)	375	473	532	519	588	88.89	0.12	0.70
Cis-vaccenic (C18:1 cis-11)	311	270	271	217	264	34.58	0.21	0.37
Oleic (C18:1 n-9)	13,181	11,427	12,667	10,021	11,417	1477	0.32	0.67
	Polyunsaturated fatty acids (PUFA)							
Linoleic (C18:2 n-6)	1182	1175	1538	1344	1329	65.15	0.04	0.03
CLA ^2^	91.9	101	135	102	133	17.99	0.17	0.73
CLA (C18:2 trans-10 cis-12)	10.7	10.5	11.4	12.1	10.5	1.10	0.74	0.47
α-linolenic (C18:3 n-3)	87.8	72.7	73.3	73.6	83.1	9.79	0.79	0.21
Arachidonic (C20:4 n-6)	293	277	337	311	265	25.88	0.79	0.16
Eicosapentaenoic (EPA; C20:5 n-3)	57.3	45.7	71.4	45.4	36.1	10.24	0.22	0.25
Docosapentaenoic (DPA; C22:5 n-3)	81.3	69.7	77.1	74.7	61.9	9.43	0.29	0.74
Docosahexaenoic (DHA; C22:6 n-3)	19.1	16.1	20.1	18.4	14.0	2.47	0.34	0.38

^1^ L = linear; Q = quadratic; significant at *p* < 0.05; CLA, conjugated linoleic acid (C18:2 cis-9 trans-11/trans-7 cis-9); ^2^ coeluted peak with C18:2 trans-7 cis-9 and C18:2 cis-9 trans-11.

**Table 5:** Fatty acid groups, sums (Ʃ), ratios, and nutritional quality of lipid fraction (mg/kg of meat) of the *Longissimus lumborum* muscle of feedlot lambs fed diets with whole corn germ (WCG).

Item ^1^	Inclusion Level of WCG (g/kg DM)					SEM	*p*-Value ^2^	
	0	30	60	90	120		L	Q
SFA	13,552	12,352	14,454	12,204	12,926	1114	0.70	0.90
MUFA	14,714	12,835	14,277	11,365	13,014	1592	0.36	0.66
PUFA	1824	1768	2263	1982	1933	92.04	0.17	0.04
BCFA	146	107	158	132	120	16.52	0.63	0.74
MUFA:SFA	1.1	1.0	0.9	0.9	1.0	0.05	0.15	0.27
PUFA:SFA	0.13	0.15	0.16	0.17	0.15	0.01	0.31	0.27
DFA	21,637	19,256	22,694	18,300	19,987	1847	0.49	0.96
Neutral	5165	4713	6202	5018	5106	375	0.88	0.28
Total	32,576	29,229	33,464	27,733	30,206	2800	0.51	0.87
Ʃ-6	385	378	472	413	398	30.40	0.55	0.17
Ʃ-3	158	131	169	138	112	17.17	0.15	0.32
omega-6:omega-3	2.5	3.1	2.9	3.1	3.6	0.30	0.03	0.90
Atherogenicity index	0.6	0.6	0.5	0.6	0.6	0.02	0.54	0.19
Thrombogenicity index	1.6	1.7	1.7	1.8	1.7	0.07	0.15	0.40
Hypocholesterolemic (h)	14,738	12,950	14,620	11,752	13,073	1454	0.35	0.76
Hypercholesterolemic (H)	8319	8238	8101	7009	7736	977	0.43	0.85
h:H index	1.9	1.8	1.9	1.8	1.8	0.05	0.26	0.63
Δ^9^-desaturase C16	7.8	7.1	7.0	7.1	7.0	0.37	0.14	0.42
Δ^9^-desaturase C18	71.3	70.0	66.8	67.0	68.6	2.07	0.24	0.26
Elongase	70.6	70.1	71.6	70.7	70.0	0.74	0.77	0.28

^1^ SFA, saturated fatty acid; MUFA, monounsaturated fatty acid; PUFA, polyunsaturated fatty acid; DFA, desirable fatty acids; Ʃ-6, sum of omega-6 fatty acids; Ʃ-3, sum of omega-3 fatty acids; h:H, hypocholesterolemic to hypercholesterolemic FA ratio (h:H) index; ^2^ L = linear; Q = quadratic; significant at *p* < 0.05.

## Data Availability

No new data were created or analyzed in this study. Data sharing is not applicable to this article.
